# Ultrasonographic assessment of ocular parameters in dogs: effects of weight and breed, controlled for BCS and age

**DOI:** 10.3389/fvets.2024.1482948

**Published:** 2024-11-12

**Authors:** Yourang Kim, Bumseok Kim, Kichang Lee, Hakyoung Yoon

**Affiliations:** ^1^Department of Veterinary Medical Imaging, College of Veterinary Medicine, Jeonbuk National University, Iksan, Republic of Korea; ^2^VIP Animal Medical Center, Seoul, Republic of Korea; ^3^Biosafety Research Institute and College of Veterinary Medicine, Jeonbuk National University, Iksan, Republic of Korea; ^4^Institute of Animal Transplantation, Jeonbuk National University, Iksan, Jeollabuk-do, Republic of Korea

**Keywords:** canine, LEL:GAL ratio, ocular parameter, reference range, ultrasound

## Abstract

**Introduction:**

Ophthalmic ultrasound is useful tool for examining canine eyes. Previous studies have compared ocular parameters using ophthalmic ultrasonography based on body weight, breed, sex, and age. However, there are limited studies involving large numbers of dogs with controlled body condition score. Furthermore, to the authors' knowledge, there are no established parameters that can be used independently of body weight. The aim of this study was (1) to compare ultrasonography ocular parameters based on body weight, breed, sex, and age in dogs with an ideal body condition score, (2) to establish normal reference ranges for ultrasonography ocular parameters on a large number of samples, (3) to establish an ultrasonography ocular parameter ratio that can be used regardless of body weight.

**Methods:**

A total of 225 dogs were collected, of which 120 dogs without abnormalities on ophthalmologic and clinical examinations were included according to the inclusion criteria. The ocular parameters measured were the anterior chamber (AC), vitreous chamber (VC), lens axial length (LAL), lens equatorial length (LEL), and globe axial length (GAL).

**Results:**

In LEL and GAL, a strong positive correlation was observed with body weight, and significant differences were identified between all body weight groups (*p* < 0.05). The mean LEL for each body weight groups is as follows: 1 ≤ body weight < 5 kg; 1.118 ± 0.032 cm, 5 ≤ body weight < 10 kg; 1.17 ± 0.03 cm, 10 ≤ body weight < 20 kg; 1.218 ± 0.018 cm, 20 ≤ body weight < 35 kg; 1.313 ± 0.03 8cm (*R*^2^ = 0.820; β = 0.008; *p* < 0.001). The mean GAL for each body weight groups is as follows: 1 ≤ body weight < 5 kg; 1.731 ± 0.076 cm, 5 ≤ body weight < 10 kg; 1.841 ± 0.064 cm, 10 ≤ body weight < 20 kg; 1.915 ± 0.043 cm, 20 ≤ body weight < 35 kg; 2.027 ± 0.059 cm (*R*^2^ = 0.598; β = 0.012; *p* < 0.05). The positive correlation with body weight was weaker for the AC, VC, and LAL than for the LEL and GAL. No significant differences were found among breeds, sexes, or ages, nor between the left and right eyes in all ocular parameters (*p* > 0.05). And we found that LEL:GAL ratio has no correlation with body weight (0.642 ± 0.022; *R*^2^ = −0.006; β = 0.000; *p* > 0.05).

**Discussion:**

This study identified significant correlations between LEL, GAL, and body weight in dogs with ideal body condition. We established normal reference ranges for ocular parameters within each BW group and breed based on a large number of samples. In addition, we present the LEL:GAL ratio, which is a constant value regardless of body weight or breed, as expected to be clinically useful in ocular evaluation.

## 1 Introduction

Ophthalmic ultrasound is an inexpensive, rapid, and non-invasive method for examining the eye. Unlike computed tomography and magnetic resonance imaging, this procedure does not require anesthesia or sedation, and its effectiveness has been studied extensively (1–3). The evaluation of structures in the posterior segment of the eye is limited in the presence of an opacification of the anterior segment of the cornea or when there is an ocular problem that causes clouding of the lens, such as in cataracts (4). In such cases, ultrasound can be used to assess the posterior segment of the eye. Moreover, ultrasound is effective in evaluating conditions such as foreign bodies, tumors, inflammation, and parasites in the periocular and retrobulbar regions that are challenging to assess with an ophthalmic examination (5). Ultrasound can also be used to measure ocular parameters, which can assist in the diagnosis of eye conditions, particularly when they affect both eyes. For instance, knowledge of the normal range of eye size can help differentiate between microphthalmos and buphthalmos, where the eye is actually abnormal in size, and enophthalmos and exophthalmos, where the eye is actually normal in size but appears small or large due to the eye being recessed or protruding from the orbit (6). Furthermore, measuring ocular parameters can provide information for implant sizing decisions in ocular surgeries. For instance, in cataract surgery, measuring the equatorial length of the lens can assist in determining the size of the intraocular lens (7, 8). In this context, ocular ultrasound serves as an effective diagnostic tool in dogs, and measuring ocular parameters is important.

Several studies have been conducted to measure and compare ocular parameters according to factors, such as body weight (BW), breed, sex, and age. Specific breeds, including French bulldogs, Latvian hunting dogs, Shih Tzus, Pomeranians, Beagles, and Cocker Spaniels, have also been studied (9–15). However, previous studies using ultrasound have involved relatively small sample sizes and have not accounted for body condition score (BCS). To the best of our knowledge, there are no established ocular measurements that can be used independently of BW across a large sample size; hence, this study aimed (1) to compare ultrasonography ocular parameters based on BW, breed, sex, and age in dogs with an ideal BCS of four or five; (2) to establish normal reference ranges for ultrasonography ocular parameters on a large number of samples; and (3) to establish an ultrasonography ocular parameter ratio that can be used regardless of BW.

## 2 Materials and methods

### 2.1 Animals

This was a retrospective and observational study. Ultrasound images, medical histories, and ophthalmic examination results of 225 dogs were collected between October 2022 and July 2023.

The inclusion criteria were the following: age range of 1–9 years, BW of 2–35 kg, BCS of 4–5/9, and the absence of abnormalities on ophthalmologic and clinical examinations. To identify healthy individuals, fluorescein staining, Schirmer's tear test, tonometry, and blood analysis were performed. Of the total 225 dogs seen, 120 dogs were included in the analysis. The dogs were then classified into four groups according to BW: BW Group 1 (1 ≤ BW < 5 kg); BW Group 2 (5 ≤ BW < 10 kg); BW Group 3 (10 ≤ BW < 20 kg); and BW Group 4 (20 ≤ BW < 35 kg). They were also divided into three groups based on age: Age Group A (1 ≤ age < 3 years), Age Group B (3 ≤ age < 7 years), Age Group C (7 ≤ age < 9 years).

This study was approved by the Institutional Animal Care and Use Committee of the Jeonbuk National University, Iksan-si, Jeollabuk-do, Republic of Korea (approval no. JBNU 2023-03-007).

### 2.2 Measurements

Ocular ultrasound was performed using a 13 MHz linear transducer (Aplio 300; Canon Medical Systems, Europe B.V., Zoetermeer, Netherlands). The eyes were bilaterally examined in the horizontal plane using a linear probe and the transcorneal technique.

**Figure 1:**
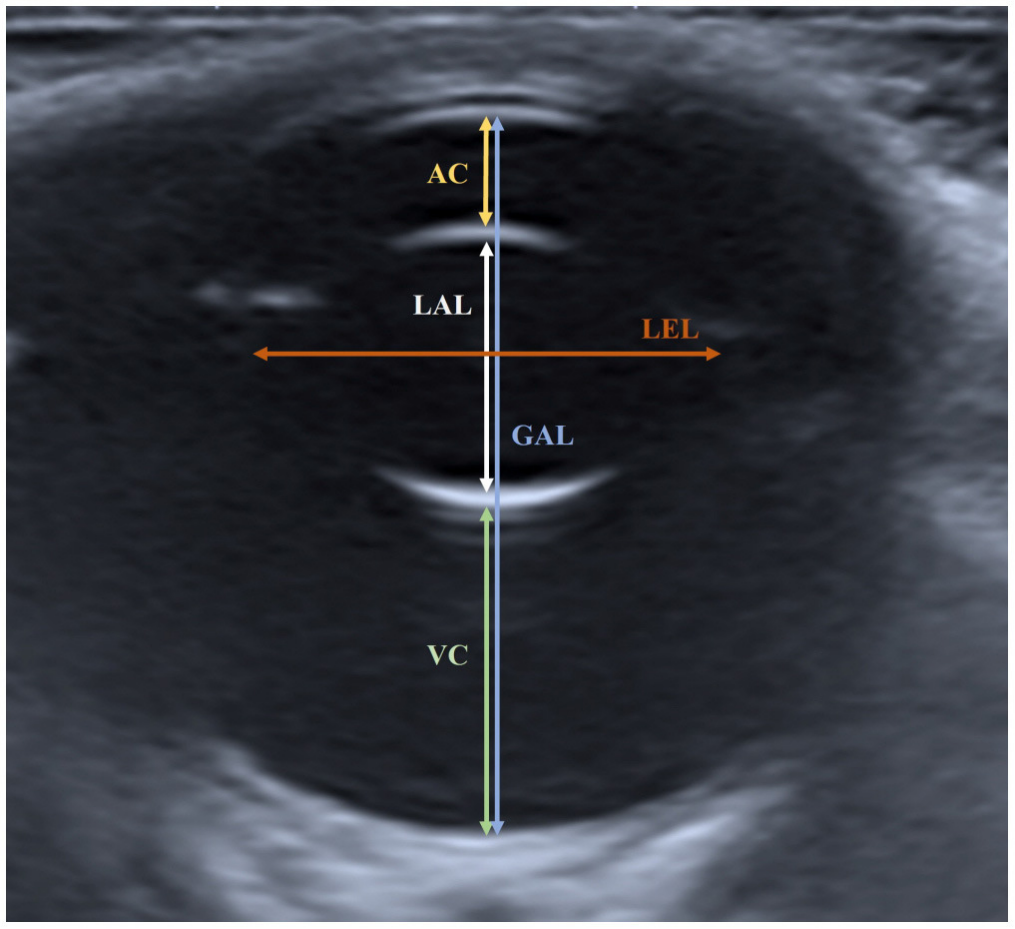
Measured ocular parameters in the horizontal plane of ocular sonogram. The AC was measured as the distance from the center of the corneal endothelium to the center of the anterior lens capsule. The VC was measured as the distance from the posterior lens capsule to the retina. The LAL was measured as the distance from the anterior lens capsule to the posterior lens capsule. The LEL was measured as the distance from one side of the lens to the other side in the equatorial plane. The GAL was measured as the distance from the corneal endothelium to the inner surface of the retina-choroid-sclera complex.

The following parameters were measured: anterior chamber (AC), vitreous chamber (VC), lens axial length (LAL), lens equatorial length (LEL), and globe axial length (GAL) (Figure 1). The AC was measured as the distance from the center of the corneal endothelium to the center of the anterior lens capsule. The VC was measured as the vertical distance from the center of the posterior lens capsule to the retina. The LAL was measured as the distance between the centers of the anterior and posterior lens capsules. The LEL was measured as the longest distance from one end of the lens to the other end at the equator. The GAL was measured as the vertical distance from the center of the corneal endothelium to the inner surface of the retina-choroid-sclera complex.

### 2.3 Statistics

Statistical analyses were performed using IBM SPSS Statistics (version 27.0; IBB Corp., Armonk, NY). All values were presented as means and standard deviations. The normality of the continuous data was tested by calculating skewness and kurtosis using descriptive statistics (16). The homogeneity of the variables was analyzed by applying the Levene test. Pearson correlation analysis was used to evaluate the relationship between body weight, age, and ocular parameters. Linear regression analysis was conducted to determine the effects of the variables on ocular parameters and to assess whether these effects were statistically significant. One-way analysis of variance (ANOVA) was used to investigate the differences in ocular parameters between BW groups, between breeds, and between age groups. When the ANOVA results were significant (*p* < 0.05), Scheffé's test was used for *post-hoc* testing. Independent *t*-tests were used to determine whether there were differences between sexes, and paired *t*-tests were used to compare the left and right eyes. Statistical significance was set at *p* < 0.05, and was considered highly significant at *p* < 0.001.

## 3 Result

A total of 120 dogs were included in the analysis, including 62 males (51.7%) and 58 females (48.3%). The mean age of all dogs was 5.67 ± 2.65 years (1–9 years) and the mean BW was 8.27 ± 7.58 kg (2–35 kg). The most common purpose for ocular ultrasound was for medical check (*n* = 128, 51.2%), followed by intraocular lens opacification (*n* = 34, 15.1%), ocular hyperemia (*n* = 18, 8%), other ocular diseases (*n* = 13, 5.8%), facial edema (*n* = 9, 4%), mass (*n* = 7, 3.1%), trauma (*n* = 6, 2.7%), foreign body (*n* = 6, 2.7%), and other problems (*n* = 4, 1.7%).

The breeds, population, and sexes of the dogs were: Maltese (*n* = 18, 13 males, five females), Poodles (*n* = 18, 11 males, seven females), Bichon Frise (*n* = 15, six males, nine females), Pomeranians (*n* = 14, nine males, five females), Chihuahuas (*n* = 11, seven males, four females), Shih Tzu (*n* = 9, five males, four females), Japanese Spitz (*n* = 10, two males, eight females), Jindo Dogs (*n* = 13, six males, seven females), Golden Retriever (*n* = 10, four male, six females), and other breeds (*n* = 2, Shetland Sheepdog, one male, and Samoyed, one female).

The ocular parameters measured for each BW group and breed group (mean ± standard deviation, 95% confidence interval) are summarized in Tables 1, 2.

**Table 1:** Mean ± SD (cm) (95% CI) of AC, VC, LAL, LEL, GAL for left and right eyes of the groups classified by BW.

**BW group**		**Group 1 (1 ≤ BW < 5 kg) (*n* = 51)**	**Group 2 (5 ≤ BW < 10 kg) (*n* = 45)**	**Group 3 ( 10 ≤ BW < 20 kg) (*n* = 12)**	**Group 4 (20 ≤ BW < 35 kg) (*n* = 12)**
BW		3.60 ± 0.76	6.97 ± 1.43	12.89 ± 2.79	28.45± 5.25
AC	OS	0.260 ± 0.024 (0.253–0.267)	0.281 ± 0.027 (0.273–0.289)	0.325 ± 0.025 (0.309–0.341)	0.347 ± 0.029 (0.328–0.365)
	OD	0.259 ± 0.025 (0.252–0.267)	0.282 ± 0.028 (0.274–0.291)	0.328 ± 0.025 (0.312–0.344)	0.346 ± 0.028 (0.327–0.364)
VC	OS	0.784 ± 0.059 (0.768–0.801)	0.910 ± 0.038 (0.886–0.934)	0.910 ± 0.038 (0.886–0.934)	0.950 ± 0.031 (0.930–0.970)
	OD	0.785 ± 0.059 (0.768–0.801)	0.913 ± 0.045 (0.885–0.942)	0.913 ± 0.045 (0.885–0.942)	0.950 ± 0.030 (0.931–0.968)
LAL	OS	0.611 ± 0.022 (0.605–0.617)	0.619 ± 0.026 (0.611–0.626)	0.618 ± 0.021 (0.604–0.631)	0.671 ± 0.022 (0.657–0.684)
	OD	0.611 ± 0.024 (0.604–0.618)	0.62 ± 0.025 (0.612–0.627)	0.616 ± 0.025 (0.6–0.632)	0.670 ± 0.021 (0.656–0.683)
LEL	OS	1.117 ± 0.032 (1.108–1.127)	1.168 ± 0.03 (1.16–1.18)	1.217 ± 0.019 (1.205–1.229)	1.314 ± 0.039 (1.289–1.339)
	OD	1.118 ± 0.033 (1.108–1.127)	1.171 ± 0.03 (1.162–1.18)	1.218 ± 0.018 (1.207–1.229)	1.311 ± 0.038 (1.287–1.335)
GAL	OS	1.730 ± 0.075 (1.709–1.751)	1.84 ± 0.066 (1.82–1.86)	1.911 ± 0.043 (1.884–1.938)	2.029 ± 0.059 (1.992–2.066)
	OD	1.732 ± 0.078 (1.71–1.754)	1.84 ± 0.063 (1.823–1.861)	1.918 ± 0.043 (1.891–1.946)	2.024 ± 0.060 (1.987–2.062)

BW, body weight; SD, standard deviation; CI, confidence interval; OS, oculus sinister; OD, oculus dexter; AC, anterior chamber; VC, vitreous chamber; LAL, lens axial length; LEL, lens equatorial length; GAL, globe axial length.

**Table 2:** Mean ± SD (cm) (95% CI) of AC, VC, LAL, LEL, GAL for left and right eyes of the groups classified by breed.

**Breed**		**Maltese (*n* = 18)**	**Poodle (*n* = 18)**	**Bichon Frise (*n* = 15)**	**Pomeranian (*n* = 14)**	**Chihuahua (*n* = 11)**	**Shih Tzu (*n* = 9)**	**Spitz (*n* = 10)**	**Jindo (*n* = 13)**	**Golden Retriever (*n* = 10)**
BW		4.49 ± 1.32	5.33 ± 1.76	5.99 ± 2.13	3.35 ± 0.62	3.57 ± 1.4	6.93 ± 1.96	7.8 ± 1.42	12.54 ± 3.54	30.14 ± 3.85
AC	OS	0.270 ± 0.022 (0.259–0.281)	0.264 ± 0.031 (0.248–0.279)	0.276 ± 0.015 (0.268–0.284)	0.253 ± 0.020 (0.241–0.264)	0.258 ± 0.03 (0.238–0.278)	0.293 ± 0.024 (0.275–0.312)	0.276 ± 0.023 (0.258–0.292)	0.335 ± 0.023 (0.32–0.348)	0.350 ± 0.027 (0.33–0.369)
	OD	0.271 ± 0.026 (0.258–0.284)	0.263 ± 0.029 (0.249–0.278)	0.278 ± 0.019 (0.267–0.289)	0.251 ± 0.023 (0.237–0.264)	0.258 ± 0.029 (0.239–0.278)	0.295 ± 0.024 (0.277–0.313)	0.275 ± 0.021 (0.259–0.29)	0.339 ± 0.022 (0.325–0.352)	0.348 ± 0.026 (0.329–0.367)
VC	OS	0.789 ± 0.057 (0.761–0.817)	0.862 ± 0.045 (0.839–0.884)	0.840 ± 0.063 (0.805–0.875)	0.755 ± 0.072 (0.714–0.796)	0.791 ± 0.059 (0.748–0.827)	0.887 ± 0.036 (0.859–0.915)	0.83 ± 0.032 (0.807–0.853)	0.917 ± 0.043 (0.891–0.943)	0.949 ± 0.033 (0.926–0.972)
	OD	0.786 ± 0.057 (0.758–0.815)	0.863 ± 0.046 (0.84–0.886)	0.839 ± 0.066 (0.803–0.876)	0.758 ± 0.074 (0.715–0.801)	0.79 ± 0.057 (0.752–0.828)	0.887 ± 0.035 (0.86–0.914)	0.834 ± 0.03 (0.813–0.856)	0.922 ± 0.045 (0.894–0.949)	0.948 ± 0.032 (0.926–0.971)
LAL	OS	0.623 ± 0.020 (0.613–0.633)	0.598 ± 0.024 (0.587–0.611)	0.609 ± 0.025 (0.595–0.623)	0.614 ± 0.012 (0.607–0.62)	0.625 ± 0.025 (0.607–0.641)	0.603 ± 0.023 (0.585–0.621)	0.64 ± 0.018 (0.627–0.653)	0.619 ± 0.023 (0.605–0.632)	0.673 ± 0.023 (0.656–0.689)
	OD	0.619 ± 0.028 (0.606–0.633)	0.600 ± 0.025 (0.589–0.613)	0.612 ± 0.025 (0.598–0.626)	0.614 ± 0.014 (0.605–0.622)	0.626 ± 0.023 (0.61–0.641)	0.6 ± 0.019 (0.585–0.615)	0.641 ± 0.014 (0.631–0.651)	0.618 ± 0.026 (0.602–0.633)	0.672 ± 0.022 (0.656–0.688)
LEL	OS	1.131 ± 0.044 (1.109–1.153)	1.148 ± 0.041 (1.128–1.168)	1.153 ± 0.033 (1.134–1.171)	1.114 ± 0.021 (1.102–1.126)	1.114 ± 0.026 (1.096–1.131)	1.163 ± 0.046 (1.128–1.2)	1.172 ± 0.010 (1.164–1.179)	1.219 ± 0.021 (1.206–1.232)	1.324 ± 0.026 (1.308–1.346)
	OD	1.132 ± 0.044 (1.11–1.154)	1.149 ± 0.040 (1.129–1.169)	1.156 ± 0.034 (1.137–1.175)	1.114 ± 0.023 (1.1–1.127)	1.115 ± 0.027 (1.096–1.133)	1.172 ± 0.053 (1.131–1.213)	1.171 ± 0.013 (1.161–1.18)	1.219 ± 0.019 (1.208–1.231)	1.324 ± 0.023 (1.307–1.341)
GAL	OS	1.757 ± 0.066 (1.725–1.79)	1.799 ± 0.075 (1.762–1.837)	1.803 ± 0.086 (1.756–1.851)	1.691 ± 0.088 (1.641–1.742)	1.752 ± 0.096 (1.688–1.816)	1.853 ± 0.065 (1.804–1.903)	1.838 ± 0.028 (1.818–1.856)	1.929 ± 0.060 (1.893–1.966)	2.034 ± 0.060 (1.991–2.077)
	OD	1.761 ± 0.065 (1.728–1.793)	1.802 ± 0.077 (1.764–1.84)	1.811 ± 0.089 (1.762–1.861)	1.694 ± 0.090 (1.642–1.746)	1.752 ± 0.099 (1.685–1.818)	1.848 ± 0.061 (1.801–1.894)	1.839 ± 0.037 (1.812–1.865)	1.932 ± 0.058 (1.897–1.967)	2.029 ± 0.062 (1.985–2.073)

BW, body weight; SD, standard deviation; CI, confidence interval; OS, oculus sinister; OD, oculus dexter; AC, anterior chamber; VC, vitreous chamber; LAL, lens axial length; LEL, lens equatorial length; GAL, globe axial length.

### 3.1 Correlation between the ocular parameters and BW

**Figure 2:**
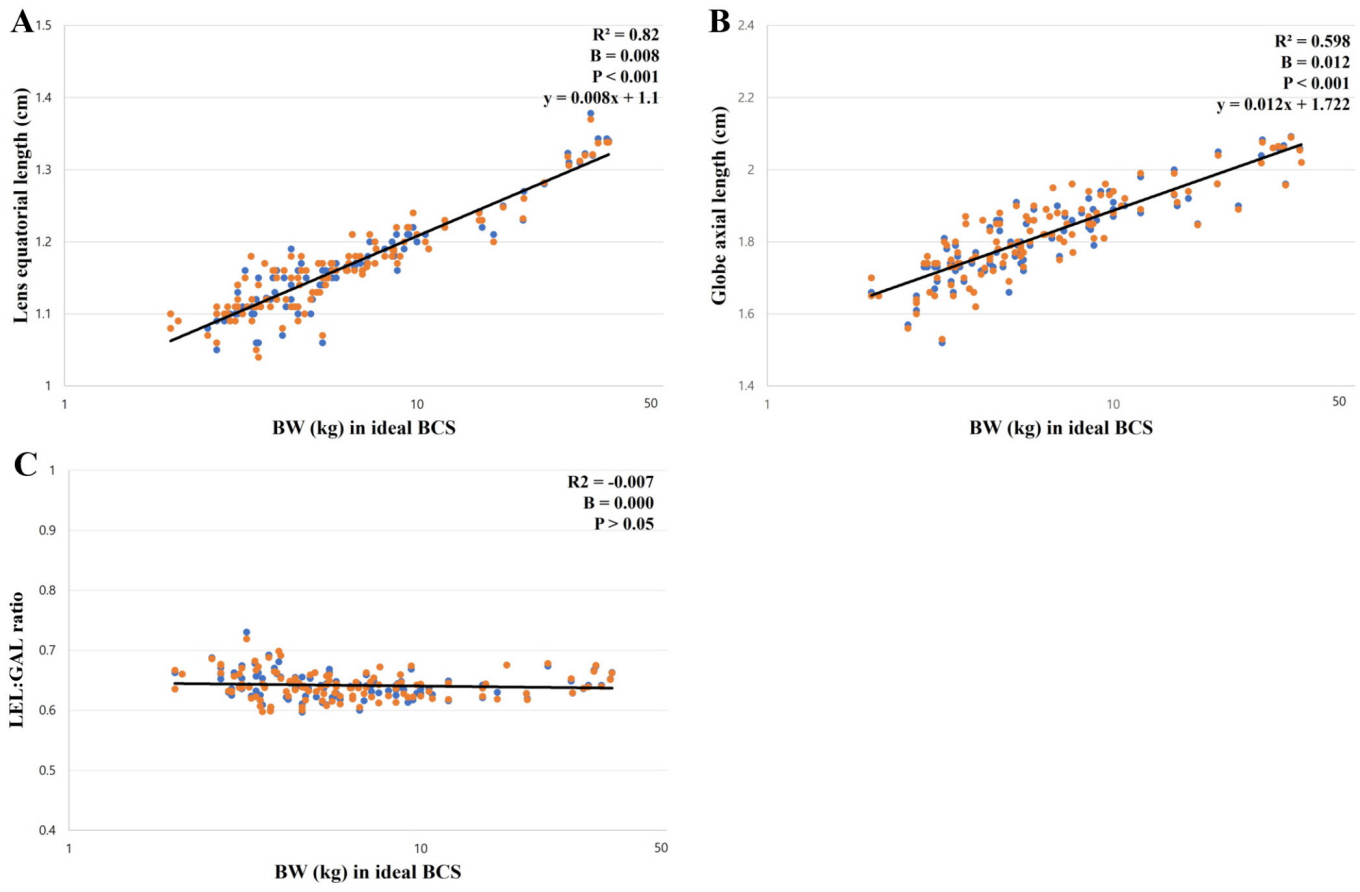
The correlation between ocular parameters and BW; **(A)** between the LEL and BW, **(B)** between the GAL and BW, **(C)** between the GAL:LEL ratio and BW. The LEL showed a linear positive correlation with the BW (*p* < 0.001; **A**). The GAL showed a linear positive correlation with the BW (*p* < 0.001; **B**). The GAL:LEL ratio did not show a correlation with the BW (*p* > 0.05; **C**).

A positive correlation was observed between the BW and all ocular parameters (*p* < 0.001). The positive correlations between the LEL, GAL, and BW were significantly higher than those between the AC, VC, and LAL. This can be identified by the high Pearson correlation coefficient and *R*^2^ values for the LEL and GAL (Table 3). The regression formula between the BW, LEL, and GAL is as follows: LEL (cm) = 0.008 × BW + 1.1 (*R*^2^ = 0.820) and GAL (cm) = 0.012 × BW + 1.722 (*R*^2^ = 0.598) (Figures 2A, B). There was no correlation between the BW and GAL:LEL ratio (1.56 ± 0.05; *R*^2^ = −0.006; β = 0.000; *p* > 0.05; Figure 2C).

**Table 3:** Pearson correlation coefficient, R square of the mean value of the left and right eye parameters with BW.

	**Pearson correlation coefficient**	***R*-square (*R*^2^)**
AC	0.706^**^	0.494
VC	0.650^**^	0.417
LAL	0.327^**^	0.347
LEL	0.906^**^	0.820
GAL	0.775^**^	0.598
LAL:GAL ratio	−0.380^**^	0.144
LEL:GAL ratio	0.041	−0.007

BW, body weight; AC, anterior chamber; VC, vitreous chamber; LAL, lens axial length; LEL, lens equatorial length; GAL, globe axial length; *p* < 0.001^**^ were considered significant.

### 3.2 Comparison of ocular parameters between the BW groups

**Figure 3:**
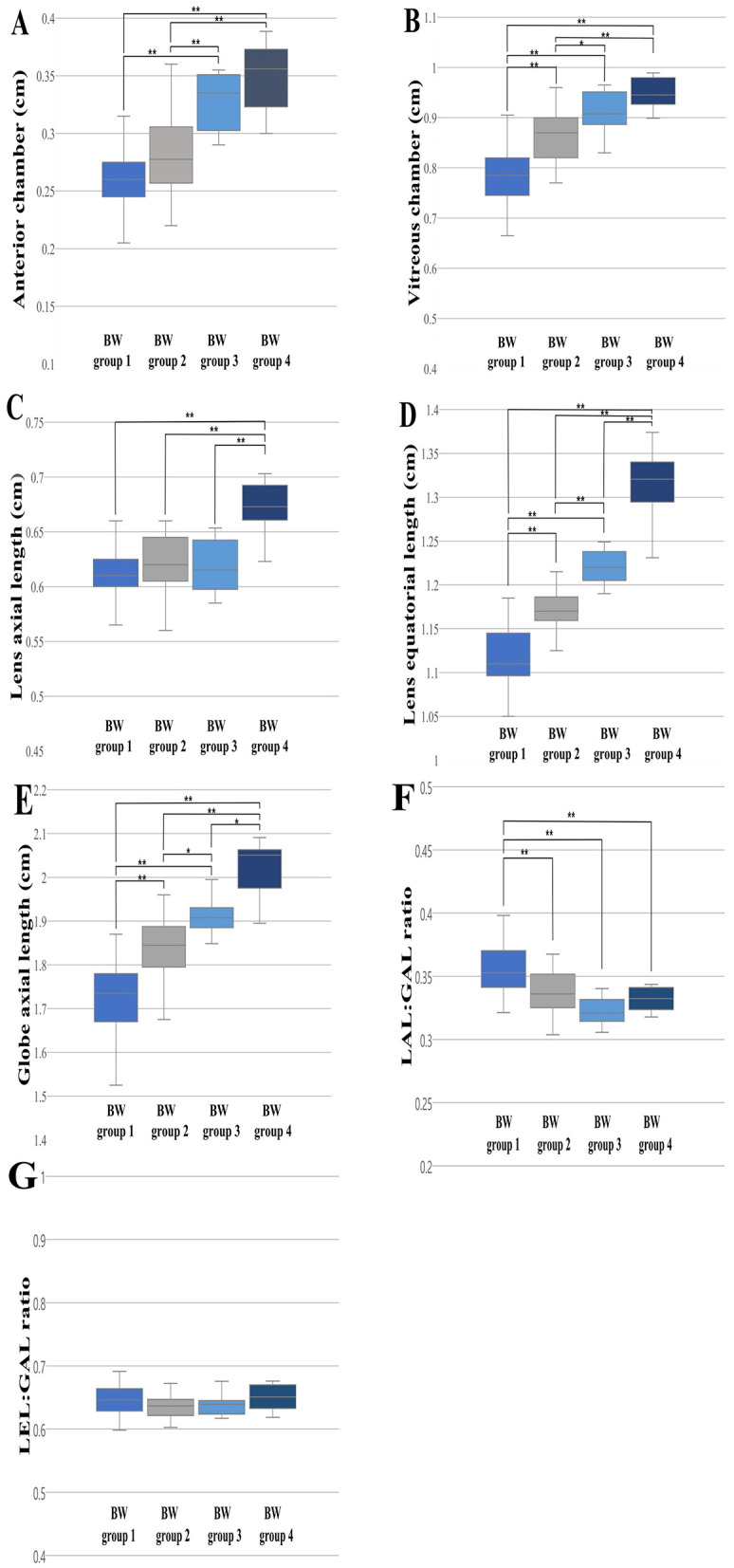
The differences in the ocular parameters for the different BW groups; **(A)** AC, **(B)** VC, **(C)** LAL, **(D)** LEL, **(E)** GAL, **(F)** LAL:GAL ratio, **(G)** LEL:GAL ratio. The LEL and GAL were statistically different between all the BW groups **(D, E)**. A statistically significant difference in the LAL:GAL ratio was identified in BW group 1 in comparison to the other BW groups **(F)**. The LEL:GAL ratio did not show significant differences among all BW groups (*p* > 0.05; **G**). *p* < 0.05*, *p* < 0.001** were considered significant.

The 120 dogs were divided into four BW groups: BW Group 1 (1 ≤ BW < 5 kg, *n* = 51); BW Group 2 (5 ≤ BW < 10 kg, *n* = 45); BW Group 3 (10 ≤ BW < 20 kg, *n* = 12); BW Group 4 (20 ≤ BW < 35 kg, *n* = 12). The ANOVA analysis and Scheffe's test showed significant differences between all the BW groups for the LEL (Group 1, 1.118 ± 0.032; Group 2, 1.17 ± 0.03; Group 3, 1.218 ± 0.018; Group 4, 1.313 ± 0.038; *p* < 0.001) and GAL (Group 1, 1.731 ± 0.076; Group 2, 1.841 ± 0.064; Group 3, 1.915 ± 0.043; Group 4, 2.027 ± 0.059; *p* < 0.05) (Figures 3D, E). For AC and VC, statistically significant differences were observed in the majority of cases, with the exception of a few BW groups. Even in instances where no statistically significant differences were identified, the mean values generally tended to increase from group 1 to group 4. In AC, the comparative analysis revealed statistically significant differences between BW groups, except for the comparisons between BW groups 1 and 2 and between BW groups 3 and 4 (Figure 3A); and in VC, significant differences were observed between the BW groups, except for between BW groups 3 and 4 (Figure 3B). For LAL, only BW group 4 showed a statistically significant difference from the other groups (Figure 3C). The LAL:GAL ratio was found to be significantly higher in BW group 1 compared to the other BW groups. However, no significant difference was observed among BW groups 2, 3, and 4 (Figure 3F). There were no statistically significant differences in the LEL:GAL ratio among all BW groups (*p* > 0.05; Figure 3G).

### 3.3 Comparison of ocular parameters between the breed groups

**Figure 4:**
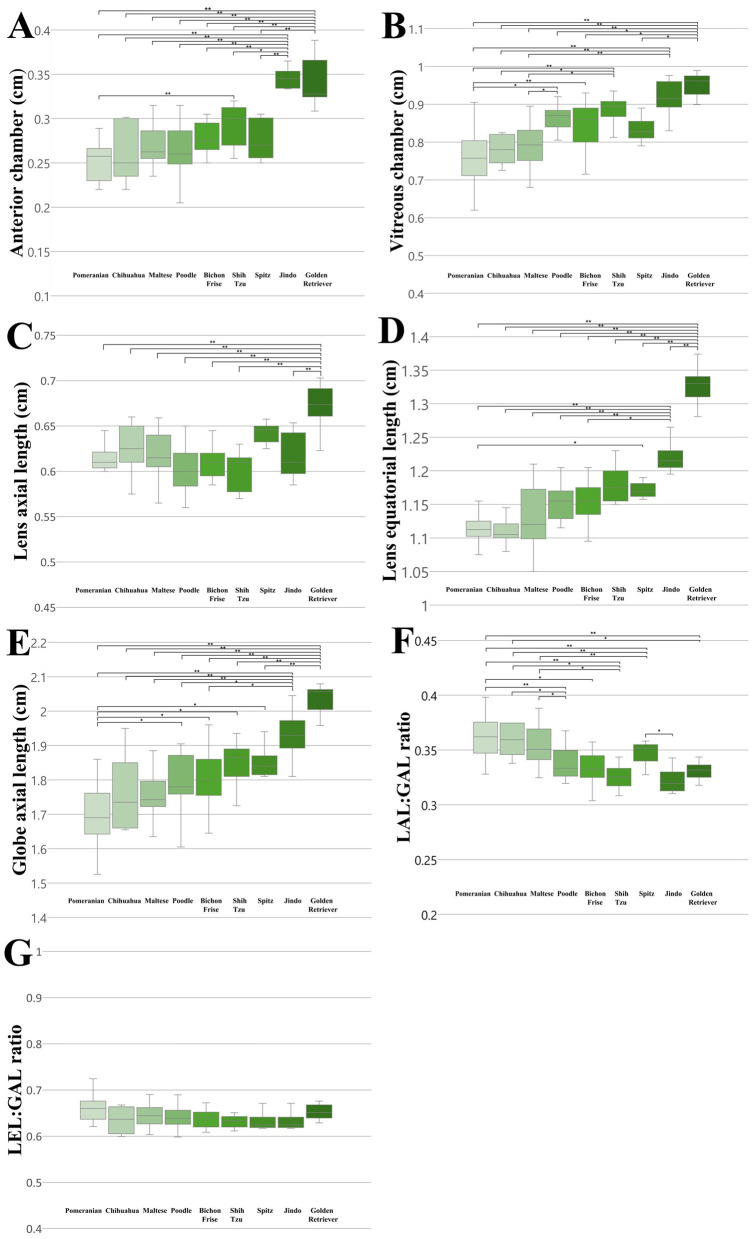
The differences in the ocular parameters for different breed groups within all BW groups; **(A)** AC, **(B)** VC, **(C)** LAL, **(D)** LEL, **(E)** GAL, **(F)** LAL:GAL ratio, **(G)** LEL:GAL ratio. **(B)** within BW group 1, **(C)** within BW group 2. In AC, VC, LEL, and GAL, the Golden Retriever and Jindo dog breeds demonstrated a higher value than other breed groups **(A, B, D, E)**. In LAL, the Golden Retriever breeds demonstrated greater values than other breed groups **(C)**. The LAL:GAL ratio was found to be elevated in the Pomeranian, Chihuahua, and Maltese breeds relative to other breeds **(F)**. In contrast, no significant differences were observed in the LEL:GAL ratio between breeds **(G)**. *p* < 0.05*, *p* < 0.001** were considered significant.

**Figure 5:**
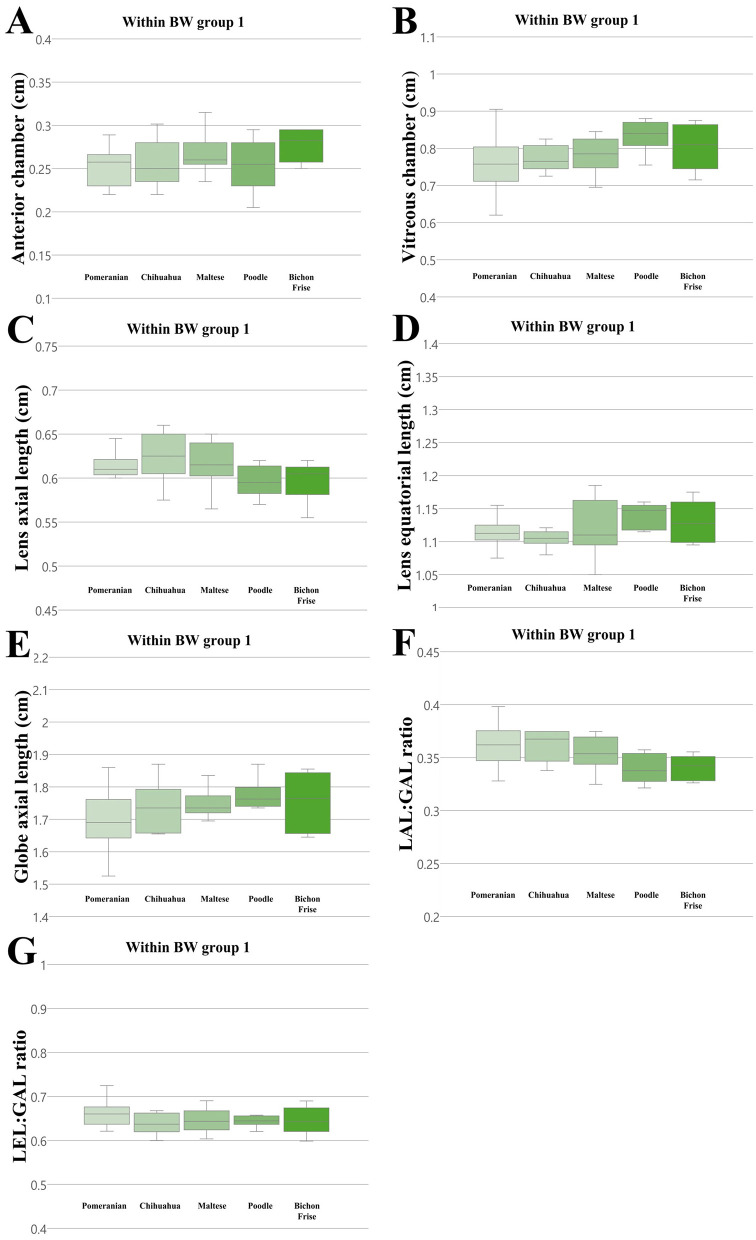
The differences in the ocular parameters for different breed groups within all BW group 1; **(A)** AC, **(B)** VC, **(C)** LAL, **(D)** LEL, **(E)** GAL, **(F)** LAL:GAL ratio, **(G)** LEL:GAL ratio. No significant differences between breeds were observed within BW groups 1 in all ocular parameters (*p* > 0.05).

**Figure 6:**
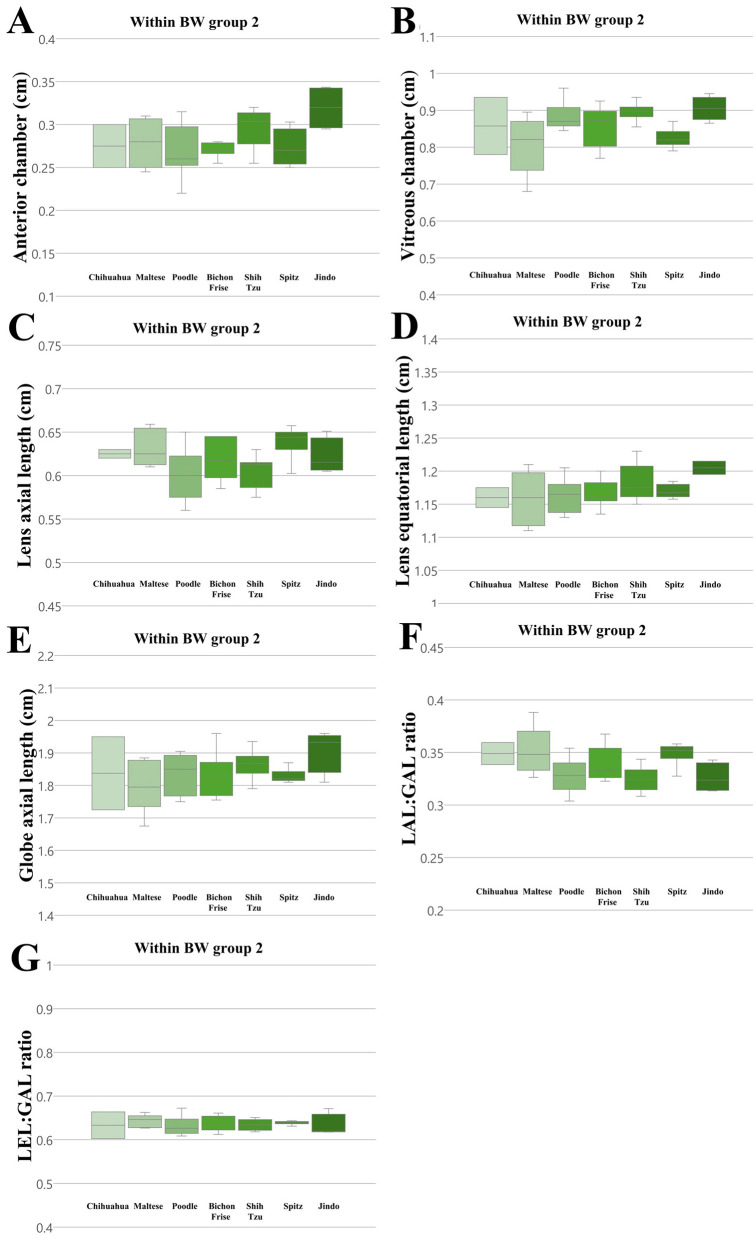
The differences in the ocular parameters for different breed groups within all BW group 2; **(A)** AC, **(B)** VC, **(C)** LAL, **(D)** LEL, **(E)** GAL, **(F)** LAL:GAL ratio, **(G)** LEL:GAL ratio. No significant differences between breeds were observed within BW groups 2 in all ocular parameters (*p* > 0.05).

We compared all ocular parameters across nine breeds, namely Pomeranian, Chihuahua, Maltese, Poodle, Bichon Frise, Shih Tzu, Japanese Spitz, Jindo Dog, and Golden Retriever (Figures 4–6). The results indicate that, when compared within all BW groups, the Golden Retriever and Jindo Dog breeds tended to exhibited greater ocular parameters than the other breeds in AC, VC, LEL, and GAL. In LAL, the Golden Retriever breeds exhibited greater ocular parameters than the other breeds as well (Figures 4A–E). However, when comparing breeds within BW groups 1 and 2, no significant differences were observed between breeds in all ocular parameters (*p* > 0.05; Figures 5A–E, 6A–E).

Additionally, the LAL:GAL ratio was found to be higher in the Pomeranian, Chihuahua, and Maltese breeds than the other breeds when compared within all BW groups (Figure 4F). However, no significant differences were observed between the breeds when they were compared within BW groups 1 and 2 (Figures 5F, 6F). This result suggests that the difference is only apparent at the general population level, but not within more specific groups.

In contrast, the LEL:GAL ratio demonstrated no significant differences between breeds when comparing within all BW groups and when comparing within BW groups 1 and 2 (Figures 4G, 5G, 6G).

### 3.4 Comparison of ocular parameters between the sexes and between the left and right eyes

The 120 dogs were divided into two groups: male (*n* = 62) and female (*n* = 58). No significant differences were found between the sexes in the independent *t*-test for any parameter (*p* > 0.05). Additionally, there were no significant differences between the left and right eyes in the paired *t*-test for any parameter (*p* > 0.05).

### 3.5 Correlation between the ocular parameters and age

The 120 dogs were divided into three age groups: Age Group A (1 ≤ age < 3 years, *n* = 28), Age Group B (3 ≤ age < 7 years, *n* = 42), Age Group C (7 ≤ age < 9 years, *n* = 50). Pearson correlation analysis revealed that there was no statistically significant correlation between age and any of the ocular parameters (*p* > 0.05). Additionally, ANOVA analysis demonstrated that there were no notable differences between age groups A, B, and C in all ocular parameters (*p* > 0.05).

## 4 Discussion

Ocular ultrasound is a valuable tool for assessing the eye when direct ophthalmic examination is difficult and can measure a variety of ocular parameters. This study establishes normal reference ranges of ocular parameters based on weight and breed, as well as ocular parameters that can be applied independently of BW.

Several studies have examined ocular parameters in relation to factors such as BW, breed, sex, and age. In a previous CT study of 100 dogs, the eyes of large breeds were significantly larger than those of medium and small breeds (*p* < 0.01), and the eyes of medium breeds were significantly larger than those of small breeds (*p* < 0.01). A correlation has been reported between eye size including eye length and BW (6). Another CT study involving 22 dogs found a statistically significant correlation between BW and globe volume (*p* = 0.003) (17). While these studies utilized BW for comparison, they did not account for BCS. It is crucial to include dogs with an ideal BCS, as neglecting this factor can lead to misinterpretation of results. Specifically, it can be difficult to differentiate weight gain due to increased adipose tissue from weight gain attributable to the actual size of the dog (18–22). Consequently, this study included dogs with a BCS of 4–5/9 and compared their ocular parameters with BW. This study is distinct from previous studies in that it employed ultrasonography, included a large sample size of 120 dogs, and subdivided the BW groups according to BCS-controlled BW.

In veterinary medicine, breed-specific ocular parameters have been studied in several dog breeds, including the French Bulldog (12), Latvian Hunting Dog (11), Shih Tzu (10, 15), Pomeranian (13), Beagle (14), and Cocker Spaniel (9). Additionally, there are few studies that have compared ocular parameters between different breeds. In a previous ultrasonographic study in dogs, the axial globe length of dolichocephalic dogs (2.12 ± 1.3 cm) was longer than mesocephalic dogs (1.99 ± 1.2 cm) (23). Other studies have indicated that dolichocephalic dogs have larger vitreous chambers and longer axial eyes than mesocephalic dogs. In brachycephalic dogs, no differences in intraocular measurements were identified compared to those in mesocephalic and dolichocephalic dogs (24). The lack of significant inter-group variation between brachycephalic and dolichocephalic dogs suggests that the relationship between skull shape and ocular metrics is relatively weak. In addition, other studies have found no significant differences in eye dimensions based on skull shape (17) and type (6). A comparable study of human subjects revealed that the axial length distribution in emmetropic children did not differ between European Caucasians and East Asians (25). In this study, when comparing across all BW groups, significant differences between breeds in ocular parameters were observed. However, given the observed differences in BW between breeds, it was assumed that differences in BW might have influenced the results. To rule this out, comparisons were made at similar BW, and no significant differences in ocular parameters were observed between breeds. In consideration of the significant positive correlation between BW and ocular parameters found in this study, it is suggested that BW, rather than breed, influences ocular parameters.

In humans, sex differences in ocular parameters have been reported. A significant association exists between height and axial length, indicating that taller individuals tend to have longer axial lengths (26–29). The mean axial length and anterior chamber depth were greater in boys than in girls (26, 27). However, in veterinary medicine, previous studies have not found any significant sex differences in dogs (1, 3, 17). This study found no significant differences between the sexes (*p* > 0.05), which is consistent with the results of previous studies. The difference between dogs and humans is thought to be related to the difference in the average height and weight between the sexes in humans, whereas there is little variation between the sexes in dogs.

Previous studies have demonstrated that in dogs between 2 weeks and 1 year of age, GAL correlates with age, showing the most rapid increase observed between 2 and 9 weeks of age, followed by a very small increase up to ~20 weeks of age (30). The depth of the eye, anterior chamber, vitreous body, and lens also display postnatal growth in puppies (31). In addition, the incidence of cataracts, which can alter ocular biometric data, is known to increase with age (32, 33). The age of 50% cataract prevalence was 9.4 years (9.4 ± 3.3 years) (34). To exclude the effect of age on ocular parameters, we selected adult dogs between 1 and 9 years of age with no ocular abnormalities. No correlation was found between any of the ocular parameters and age in adult dogs (*p* > 0.05). These results were similar to those reported in previous studies (7).

While previous studies using ultrasound were conducted on smaller sample sizes of 20–30 dogs, this study involved a larger cohort of 120 dogs. This study established a reference range for each BW group and breed, which is expected to be more accurate and specific than those reported in previous studies. To the best of our knowledge, there is no established ocular parameter that can be used regardless of the BW. In this study, the LEL:GAL ratio was found to be uncorrelated with BW (0.642 ± 0.022; *R*^2^ = −0.006; β = 0.000; *p* > 0.05), and ANOVA and *post-hoc* analyses confirmed that this ratio was not significantly different between the BW groups and between breed groups. This suggests that the LEL:GAL ratio is a constant value, independent of the BW and breed, and is expected to be a clinically useful indicator.

This study had some limitations. The number of dogs in the Groups 3 and 4 was smaller than that in the Groups 1 and 2. Additionally, this comparison was performed only for the horizontal plane and not for the vertical plane. Further studies are required to address these limitations. In canine ultrasound imaging, the full margin of the lens may not be clearly visible in certain instances. To compensate for this limitation, methods such as swept-source optical coherence tomography (OCT) and very high frequency ultrasound biomicroscopy (UBM) can be applied in humans. These imaging techniques utilize built-in programs or semi-automated measurements to more accurately identify the anterior and posterior boundaries of the lens, enabling reproducible measurements of human ocular lens parameters (35, 36). In dogs, previous studies have employed OCT to assess retinal and optic nerve morphology or choroidal vascular layer thickness (37, 38). However, no studies have evaluated canine lenses using these techniques. This is because it is challenging to adapt software designed for human ocular anatomy to provide accurate quantitative values in animals (37). Further improvements in this respect are expected to be beneficial in the evaluation of canine lens parameters. Recent studies employing scanning electron microscopy have demonstrated a positive correlation between lens thickness and the number of ciliary processes, which exhibited variation based on the head structure. Additionally, a depression on the posterior surface of the lens has been observed in brachycephalic dogs (39). As these features are difficult to assess through ultrasound, further studies that combine scanning electron microscopy and ultrasound will enhance the complementarity of the evaluation.

In conclusion, this study found significant correlations between the LEL, GAL and BW in the dogs with ideal BCS. Normal reference ranges of ocular parameters were established for each BW group and breed. In addition, a LEL:GAL ratio of 0.642 ± 0.022 (95% confidence interval: 0.639–0.654) was established, which can be used regardless of BW or breed. These results are expected to be useful in the evaluation of the eye, such as determining the size of implants in ocular surgery and assessing eye diseases that affect eye size.

## Data Availability

The raw data supporting the conclusions of this article will be made available by the authors, without undue reservation.
